# Pharmacokinetic comparison of ezetimibe/rosuvastatin/telmisartan 10/20/80 mg fixed-dose combination versus coadministration of separate tablets in healthy participants

**DOI:** 10.1007/s00210-025-04903-4

**Published:** 2026-01-08

**Authors:** Seoyoung Goh, Ki Young Huh, SeungHwan Lee, Joo-Youn Cho, Kyung-Sang Yu

**Affiliations:** Department of Clinical Pharmacology and Therapeutics, Seoul National University College of Medicine and Hospital, Seoul, Republic of Korea

**Keywords:** Fixed-dose combination, Pharmacokinetics, Bioequivalence, Dyslipidemia, Hypertension

## Abstract

This study aimed to evaluate the pharmacokinetics (PKs) for fixed-dose combination (FDC) of ezetimibe/rosuvastatin/telmisartan 10/20/80 mg (test) compared to separate tablets of ezetimibe/rosuvastatin 10/20 mg FDC and telmisartan 80 mg (reference). A randomized, open-label, 4-period, 2-sequence replicated crossover study was conducted in healthy participants. Each treatment group received a single oral dose of test or reference drug in each period. PK samples during the 1st and 2nd periods were collected for all three drugs, while only telmisartan concentration was measured in the 3rd and 4th periods to evaluate intra-subject variability (ISV) of telmisartan. Geometric mean ratios (GMRs) of PK parameters and their 90% confidence intervals (CIs) were calculated with a linear mixed effect model between each treatment group. To account for the high ISV of telmisartan’s peak plasma concentration (*C*_max_), the scaled average bioequivalence (SABE) approach was considered in this analysis. A total of 58 participants were randomized, and 49 participants completed the study. PK profiles were comparable between treatments. The GMR and 90% CIs (test-to-reference) of area under the time-concentration curve and *C*_max_ were 0.9835 (0.9260–1.0446) and 0.9873 (0.8830–1.1039) for free ezetimibe, and 0.9937 (0.9459–1.0440) and 1.0617 (0.9844–1.1415) for total ezetimibe. For rosuvastatin, corresponding values were 0.9465 (0.8883–1.0086) and 0.9062 (0.8135–1.0096); for telmisartan, 0.9728 (0.9315–1.0159) and 0.9724 (0.8846–1.0690), respectively. The result established equivalence without utilizing the SABE approach, although the ISV of telmisartan’s *C*_max_ was 40.7%. A FDC of ezetimibe/rosuvastatin/telmisartan 10/20/80 mg demonstrated comparable pharmacokinetic profiles to corresponding separate treatments.

## Introduction

Due to the frequent coexistence of hypertension and dyslipidemia, combination therapy is commonly employed to improve cardiovascular outcomes (Dezii 2000). Treating hypertension and comorbid dyslipidemia with combination therapy can significantly improve cardiovascular outcomes (Kostis 2007). Statins are commonly used to treat dyslipidemia by inhibiting cholesterol synthesis in the liver (Grundy et al. 2019). Ezetimibe is often used with statins to further lower LDL cholesterol levels by reducing cholesterol absorption in the intestines, providing an additive effect (Cannon et al. 2015). Combining ezetimibe with statin therapy may reduce the need for high-dose statins and effectively lower cholesterol levels (Kim et al. 2022). Telmisartan, an angiotensin II receptor blocker, is used to treat hypertension.

Combining all three treatments, including telmisartan, into a single fixed-dose combination (FDC) would maximize the benefits of improving low adherence (Lee et al. 2024). However, polypharmacy increases the number of pills patients need to take, which can lead to lower adherence compared to monotherapy. Using FDC can increase adherence while maintaining comparable or better therapeutic effects (Bangalore et al. 2007a, b). By simplifying the treatment regimen, FDCs reduce the pill burden for patients, which is a key factor in improving adherence (Blank et al. 2005). Bangalore, Kamalakkannan et al. have shown that patients on FDC therapy have better compliance and overall health outcomes compared to those on separate medications. Additionally, FDCs can lead to a reduction in overall healthcare costs by improving adherence and reducing complications (Bangalore et al. 2007a, b).

Considering the challenges of polypharmacy, the development of a triple combination FDC addresses this issue while maintaining therapeutic efficacy. Rosuvamibe Tab. (ezetimibe 10 mg/rosuvastatin 20 mg, Yuhan Corporation, Republic of Korea) and other combination dosages of ezetimibe and rosuvastatin FDCs have been approved by the Ministry of Food and Drug Safety of the Republic of Korea (MFDS), while there has been no approval for any FDC of all three drugs. This drug combination enabled a higher proportion of patients to achieve recommended LDL-C goals compared to rosuvastatin monotherapy, without a high risk of adverse events (Kim et al. 2022).

As recommended by the European Medicines Agency (EMA) and MFDS guidance (European Medicines Agency 2010; Ministry of Food and Drug Safety 2023), a randomized, 2-period, two-sequence, two-treatment, single-dose crossover study is typically conducted to evaluate equivalence. A highly variable drug (HVD) is one that exhibits more than 30% intra-subject variability (ISV) of maximum plasma concentration (*C*_max_), indicating significant differences in plasma concentrations among individuals. Such drugs may benefit from the application of the scaled average bioequivalence (SABE) approach, which allows for a reduced sample size while maintaining sufficient power to establish bioequivalence (European Medicines Agency 2010). Telmisartan was classified as a HVD in the MFDS, exhibiting significant ISV that makes bioequivalence assessment challenging (Ministry of Food and Drug Safety 2025). Previous studies reported ISV values of telmisartan’s *C*_max_ as 42.94% and 41.96%. (Kang et al. 2018; Lee et al. 2017). In this study, a 4-period fully replicated crossover design was applied to account for the variability of telmisartan and ensure a robust evaluation of equivalence.

This study aimed to evaluate the pharmacokinetics (PKs) of an FDC containing ezetimibe/rosuvastatin/telmisartan 10/20/80 mg (test) compared to coadministration of separate tablets of ezetimibe/rosuvastatin 10/20 mg (FDC) and telmisartan 80 mg (reference).

## Methods

### Study subjects

The study was approved by the MFDS and the Institutional Review Board at H Plus Yangji Hospital. The study was registered in the Clinical Research Information Service in Korea (KCT0009387). All procedures were conducted in accordance with the Declaration of Helsinki (World Medical Association 2013) and Good Clinical Practice (International Council for Harmonisation of Technical Requirements for Pharmaceuticals for Human Use 2016). Written informed consent was obtained from all volunteers prior to any study-related procedures.

Healthy adults aged above 19 years with body mass index (BMI) between 18 and 30 kg/m^2^ were eligible for the study. Individuals with moderate to severe renal impairment, gastrointestinal conditions affecting absorption, or a history of gastrointestinal surgery were excluded. Volunteers with a history of hereditary angioedema or angioedema following angiotensin-converting enzyme inhibitors or angiotensin receptor blockers were excluded. Additionally, volunteers with a history of cyclosporine or fibrate use were excluded due to the risk of drug-drug interaction or myopathy with statin.

### Study design

**Fig. 1:**
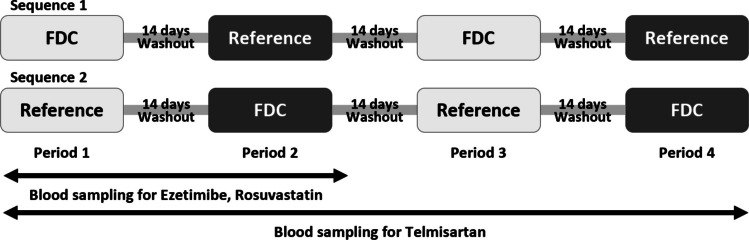
Study design. Abbreviations: FDC, fixed-dose combination; *FDC: AD-201 (ezetimibe/rosuvastatin/telmisartan 10/20/80 FDC). Reference: ezetimibe/rosuvastatin 10/20 mg FDC and telmisartan 80 mg combination tablet

A randomized, open-label, 4-period, 2-sequence replicated crossover study was conducted in healthy participants. The test drug (T) was FDC of ezetimibe 10 mg/rosuvastatin 20 mg/telmisartan 80 mg (AD-201, Addpharma Pharmaceutical Corporation, Republic of Korea), while the reference drugs (R) were the coadministration of separate tablets of ezetimibe 10 mg/rosuvastatin 20 mg FDC (Rosuvamibe Tab. 10/20 mg, Yuhan Corporation, Seoul, Republic of Korea) and telmisartan 80 mg (Micardis Tab., Boehringer Ingelheim Korea, Republic of Korea). Due to the high variability of telmisartan *C*_max_, a 4-period replicated crossover design was chosen. The SABE approach was used with expanded confidence intervals applied in cases where ISV of *C*_max_ of telmisartan exceeded 30%. The study consisted of two sequences, TRTR and RTRT. Each treatment period was separated by a 14-day washout period (Fig. 1). Each treatment group received a single oral dose of either the test or reference drugs during each period. PK samplings were conducted at pre-dose and at 0.25, 0.5, 0.75, 1, 1.25, 1.5, 2, 3, 4, 5, 6, 7, 8, 10, 12, 24, 48, and 72 h post-dose. PK samples during the 1st and 2nd periods were collected for all three drugs, while only telmisartan concentration was measured in the 3rd and 4th periods to assess ISV. Blood samples were collected in K2 EDTA tubes and centrifuged at 3000 rpm for 10 min at 4 °C. Each sample was aliquoted into 0.5 mL portions and stored in a polypropylene tube at − 70 °C until analysis.

### Bioanalytical analysis

The plasma concentrations of free ezetimibe, total ezetimibe, rosuvastatin, and telmisartan were determined using a validated liquid chromatography method on a Waters ACQUITY UPLC™ system (Waters Corporation, MA, USA) coupled with a Waters ACQUITY UPLC BEH C18 column (1.7 μm, 2.1 mm ID × 50 mm L). The Waters Xevo™ TQ-S MS (Waters Corporation, MA, USA) was selected for detecting free ezetimibe, total ezetimibe, and rosuvastatin. The Waters Micromass Quattro Premier™ XE Mass Spectrometer (Waters Corporation, MA, USA) was used for telmisartan. The free ezetimibe refers specifically to the unconjugated ezetimibe, and total ezetimibe represents the sum of unconjugated ezetimibe and its primary active metabolite, ezetimibe glucuronide.

The multiple reaction monitoring (MRM) was performed in negative ion electrospray mode for the quantification of free ezetimibe, total ezetimibe, and their internal standards (IS), ezetimibe-d_4_. The monitored transitions for free ezetimibe and its IS were 407.98 → 270.95 and 411.99 → 270.94. For total ezetimibe and its IS, the monitored transitions were 408.26 → 271.10 and 412.27 → 271.10.

Mass spectrometry under MRM using positive ion electrospray mode was performed for rosuvastatin, rosuvastatin-d_3_, telmisartan, and telmisartan-d_7_. The monitored transitions were 481.97 → 257.99 and 485.04 → 261.00 for rosuvastatin and rosuvastatin-d_3_. For telmisartan and telmisartan-d_7_, the monitored transitions were 515.30 → 276.26 and 522.30 → 280.26.

The range of linearity in the calibration curve for each analyte was 0.0500–20.0 ng/mL for free ezetimibe (*r*^2^ ≥ 0.9977), 0.500–200 ng/mL for total ezetimibe (*r*^2^ ≥ 0.9991), 0.100–100 ng/mL for rosuvastatin (*r*^2^ ≥ 0.9985), and 2.00–2000 ng/mL for telmisartan (*r*^2^ ≥ 0.9988).

A precision range of free ezetimibe, total ezetimibe, rosuvastatin, and telmisartan was 1.2–3.4%, 1.3–2.4%, 1.1–2.2%, and 1.4–2.2%. And accuracy ranges were − 6.6 to − 2.7% for free ezetimibe, − 3.9 to − 2.2% for total ezetimibe, − 4 to 2.4% for rosuvastatin, and − 4.5 to − 3.4% for telmisartan.

### Pharmacokinetic analysis

Pharmacokinetic parameters of free ezetimibe, total ezetimibe, telmisartan, and rosuvastatin were determined using noncompartmental analysis with Phoenix WinNonlin® (Version 8.4, Certara, CA, USA). The pharmacokinetic parameters included *C*_max_ and the time to reach maximum plasma concentration (*T*_max_), which were directly observed from data. Area under the concentration–time curve to the last measurable concentration (AUC_t_) was calculated by the linear trapezoidal linear interpolation method. Area under the curve from the time of dosing extrapolated to infinity based on the last observed concentration (AUC_inf_) and terminal-phase elimination half-life (*t*_1/2_) were calculated using the elimination rate constant.

### Statistical analysis

Statistical analysis was performed using SAS software (version 9.4; SAS Institute Inc., NC, USA). The study sample size was calculated based on previous studies involving healthy volunteers, considering the largest ISV of *C*_max_ for ezetimibe, rosuvastatin, and telmisartan (Migoya et al. 2006). Based on an ISV of ezetimibe’s *C*_max_ of approximately 34%, a test-to-reference (T/R) ratio of 0.97, and a significance level of 0.05, the required sample size to achieve 80% statistical power was calculated to be 44 subjects. To account for a dropout rate of approximately 23%, the final number of subjects was calculated to be 58. The ISV of telmisartan’s *C*_max_ was not considered for the primary sample size determination because the SABE approach requires a lower sample size than the conventional sample size determination.

A linear mixed-effects model was used to compare log-transformed pharmacokinetic parameters, with period, sequence, and treatment as fixed effects, and subjects nested within a sequence as a random effect. The systemic exposure of test and reference treatment was considered equivalent when the 90% confidence intervals (CIs) for the geometric mean ratios (GMRs) of *C*_max_ and AUC_t_ fell within the equivalence limits of 0.8 to 1.25. Additionally, an ISV of log-transformed *C*_max_ of telmisartan in each reference treatment was calculated. When ISV for *C*_max_ exceeded 30%, the confidence intervals were expanded, and the upper and lower limits were calculated using (1).

1 $$exp\lbrack\pm0.760\times\lbrack log-transformed\;ISV\;of\;C_{max}(\%)\rbrack$$

### Safety analysis

Routine safety assessments were conducted, including the monitoring of adverse events (AEs), physical examinations, clinical laboratory tests, electrocardiograms, and vital signs. The safety assessment included all participants who received at least one dose of any of the treatments.

## Results

### Subject disposition

A total of 58 participants were randomized, and 49 participants completed the study. Nine participants did not complete the study, with 4 withdrawing consents after dosing and 5 dropping out due to adverse events. Fifty-five subjects completed the study up to the second period, and 49 subjects completed all four periods, resulting in 55 subjects for the ezetimibe and rosuvastatin analysis and 49 for the telmisartan. The demographics of the total study participants are a mean ± standard deviation age of 31.62 ± 8.40 years, height of 172.73 ± 5.35 cm, weight of 69.89 ± 10.37 kg, and BMI of 23.39 ± 3.05 kg/m^2^, respectively. There were no statistically significant differences for demographic factors between participants in the two sequences.

### Pharmacokinetic results

**Fig. 2:**
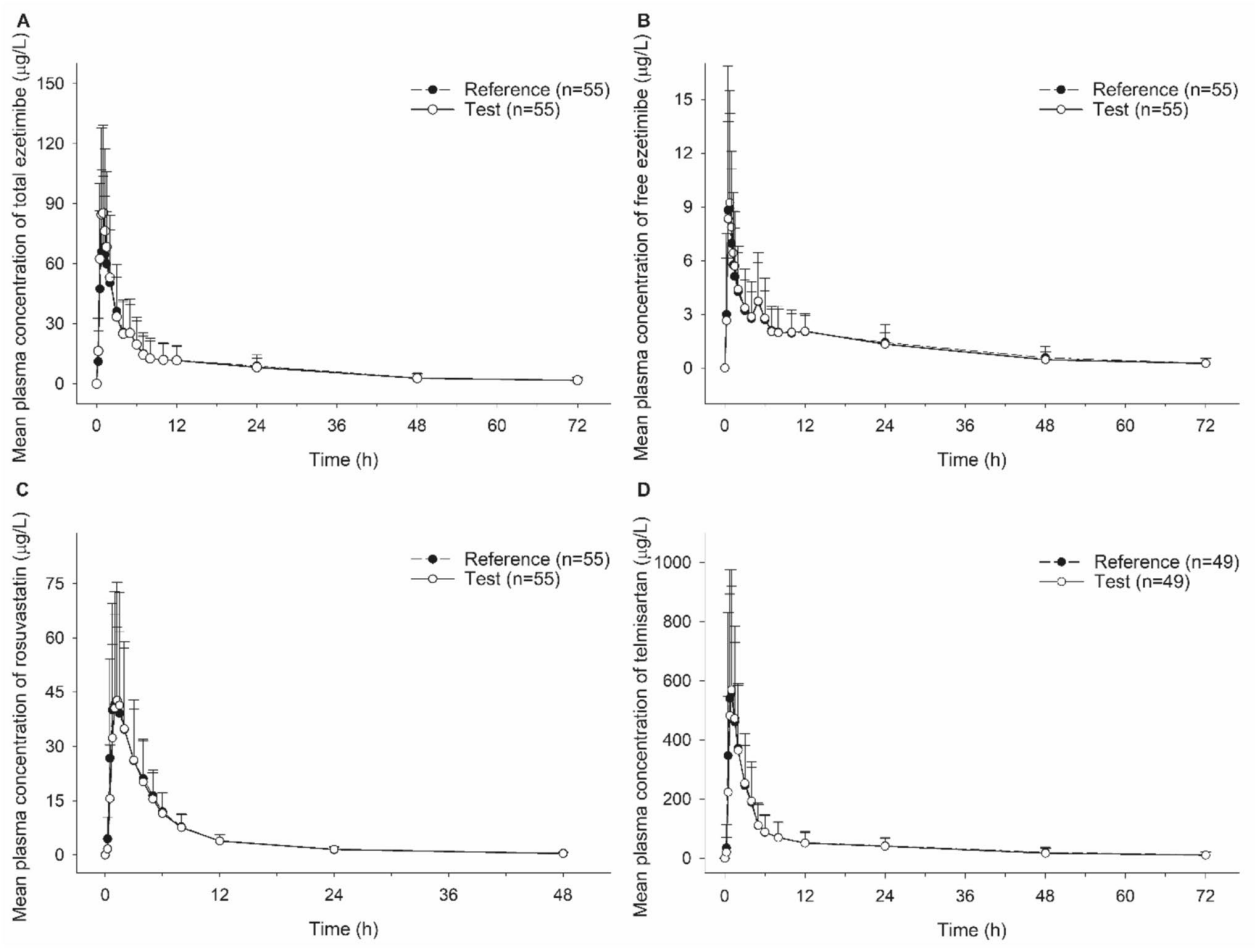
Mean plasma concentration–time profile of **A** total ezetimibe, **B** free ezetimibe, **C** rosuvastatin, and **D** telmisartan after a single oral administration of AD-201 (ezetimibe/rosuvastatin/telmisartan 10 mg/20 mg/80 mg FDC) as a test treatment or ezetimibe/rosuvastatin 10 mg/20 mg FDC and telmisartan 80 mg combination tablet as a reference. Error bars denote standard deviation. *N* denotes the number of participants included in the analysis. Abbreviations: FDC, fixed dose combination;

The PK profiles of free ezetimibe, total ezetimibe, rosuvastatin, and telmisartan demonstrated comparable absorption and elimination patterns across the FDC and separate treatments (Fig. 2).

The median value of *T*_max_ was 0.75–1.00 h in all analytes (Table 1). The GMR and 90% CIs of AUC_t_ and *C*_max_ were 0.9835 (0.9260–1.0446) and 0.9873 (0.8830–1.1039) for free ezetimibe and 0.9937 (0.9459–1.0440) and 1.0617 (0.9844–1.1415) for total ezetimibe. For rosuvastatin, the GMR and 90% CIs of AUC_t_ and *C*_max_ were 0.9465 (0.8883–1.0086) and 0.9062 (0.8135–1.0096) (Table 2) All results fell within the equivalent criteria of 0.8 to 1.25.

**Table 1:** PK parameters of total ezetimibe, free ezetimibe, rosuvastatin, and telmisartan

Variables (unit)	Total ezetimibe		Free ezetimibe		Rosuvastatin		Telmisartan	
	**Reference (*n* = 55)**	**Test (*n* = 55)**	**Reference (*n* = 55)**	**Test (*n* = 55)**	**Reference (*n* = 55)**	**Test (*n* = 49)**	**Reference (*n* = 49)**	**Test (*n* = 49)**
*T*_max_ (hr)	1.00 [0.50–3.00]	0.75 [0.50–4.00]	0.75 [0.25–5.00]	0.75 [0.25–5.00]	0.75 [0.50–4.00]	1.00 [0.50–5.00]	1.00 [0.50–3.00]	1.00 [0.50–5.00]
*C*_max_ (μg/L)	87.71 ± 35.21	94.45 ± 42.46	11.85 ± 7.16	10.97 ± 5.07	51.73 ± 29.80	50.42 ± 35.23	727.00 ± 454.59	710.16 ± 422.50
AUC_t_ (hr∙μg/L)	612.43 ± 286.11	605.35 ± 294.14	90.43 ± 48.12	87.28 ± 38.59	247.81 ± 110.83	240.94 ± 116.81	3571.90 ± 2182.73	3470.55 ± 2215.52
AUC_inf_ (hr∙μg/L)	679.35 ± 317.85	669.59 ± 325.92	99.69 ± 53.95	95.62 ± 489.67	255.14 ± 113.27	248.39 ± 118.35	3989.98 ± 2599.58	3867.78 ± 2553.32
*t*_1/2_ (hr)	21.64 ± 26.10	18.84 ± 11.23	18.40 ± 9.83	17.34 ± 9.68	10.77 ± 2.57	11.31 ± 3.01	21.72 ± 10.10	22.10 ± 12.83

All values presented are shown as mean ± standard deviation except for *T*_max_, which is presented as median [minimum–maximum]. *N* denotes the number of participants included in the analysis. *PK*, pharmacokinetic; *FDC*, fixed dose combination; *AUC*_t_, area under the concentration–time curve to the last measurable concentration; *C*_max_, maximum plasma concentration; *AUC*_*inf*_, area under the curve from zero to infinity; *T*_max_, time to reach *C*_max_; *t*_1/2_, terminal-phase elimination half-life; *Test: AD-201 (ezetimibe/rosuvastatin/telmisartan 10/20/80 FDC). Reference: ezetimibe/rosuvastatin 10/20 mg FDC and telmisartan 80 mg combination tablet

**Table 2:** Geometric mean ratios and 90% confidence intervals of free ezetimibe, total ezetimibe, rosuvastatin, and telmisartan

Drug	Variables (unit)	Geometric mean ratio* (90% confidence interval)	Intrasubject variability of reference treatment (%)
Free ezetimibe	AUC_t_ (hr∙μg/L)	0.9835 (0.9260–1.0446)	
	*C*_max_ (μg/L)	0.9873 (0.8830–1.1039)	
Total ezetimibe	AUC_t_ (hr∙μg/L)	0.9937 (0.9459–1.0440)	
	*C*_max_ (μg/L)	1.0617 (0.9844–1.1451)	
Rosuvastatin	AUC_t_ (hr∙μg/L)	0.9465 (0.8883–1.0086)	
	*C*_max_ (μg/L)	0.9062 (0.8135–1.0096)	
Telmisartan	AUC_t_ (hr∙μg/L)	0.9728 (0.9315–1.0159)	
	*C*_max_ (μg/L)	0.9724 (0.8846–1.0690)	40.7

*Geometric mean of the test treatment compared to the reference treatment. *N* denotes the number of participants included in the analysis. *AUC*_*t*_, area under the concentration–time curve to the last measurable concentration; *C*_max_, maximum plasma concentration. *Test: AD-201 (ezetimibe/rosuvastatin/telmisartan 10 mg/20 mg/80 mg FDC). Reference: ezetimibe/rosuvastatin 10 mg/20 mg FDC and telmisartan 80 mg combination tablet

**Fig. 3:**
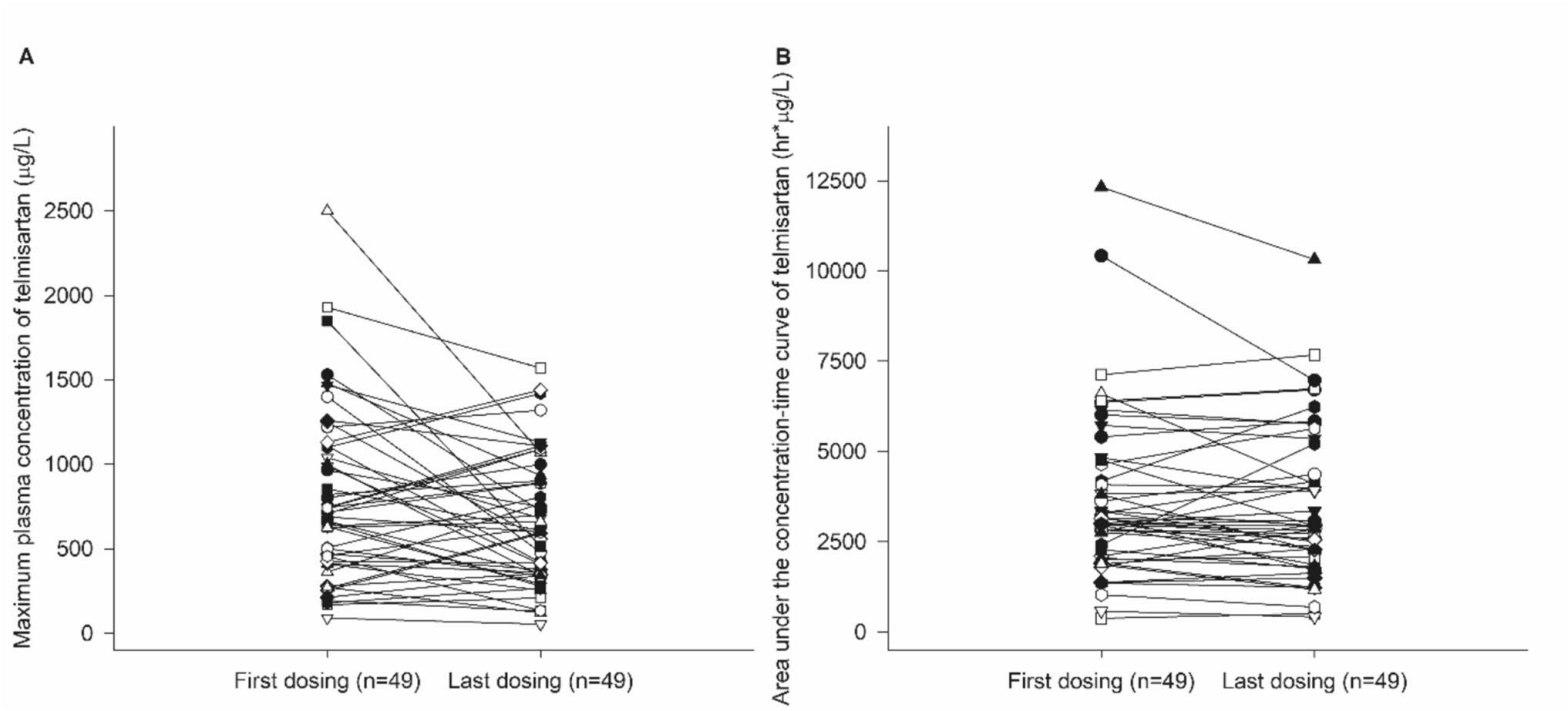
Intraindividual changes in **A** *C*_max_ and **B** AUC_t_ of telmisartan during the reference treatment. *N* denotes the number of participants included in the analysis. Abbreviations: AUC_t_, area under the concentration–time curve to the last measurable concentration; *C*_max_, maximum plasma concentration; FDC, fixed dose combination

The GMR and 90% CIs of AUC_t_ and *C*_max_ were 0.9728 (0.9315–1.0159) and 0.9724 (0.8846–1.0690) for telmisartan. The *C*_max_ of the reference treatment of telmisartan was highly variable, with an ISV of 40.7% (Fig. 3, Table 2). The 90% CIs of telmisartan could be expanded to 0.7284–1.3729. It met the equivalent criteria based on SABE as well as conventional equivalent criteria.

### Safety results

A total of 21 treatment-emergent AEs were reported by 14 subjects. Overall, most AEs were mild. A total of 4 adverse drug reactions (ADRs) were leading to one withdrawal from each of the test and reference treatments. Among these, 7 ADRs were reported in 6 participants (10.7%) following administration of the reference treatment, and 13 ADRs were reported in 11 participants (19.3%) following administration of the test treatment. One serious adverse event (SAE), a foot fracture, was assessed as unrelated to the test treatment. Four ADRs were headache, diarrhea, dizziness, and abdominal pain. All treatments were evaluated as safe and tolerable.

## Discussion

This study evaluated the pharmacokinetics and safety profiles of an ezetimibe 10 mg/rosuvastatin 20 mg/telmisartan 80 mg FDC (AD-201) compared to the coadministration of separate tablets of ezetimibe 10 mg/rosuvastatin 20 mg FDC and telmisartan 80 mg. The results demonstrated equivalence in pharmacokinetic characteristics between the test and reference treatments, and met the regulatory criteria for equivalence.

Ezetimibe undergoes rapid absorption and extensive metabolism, resulting in the formation of its major active metabolite, ezetimibe glucuronide. Approximately 80–90% of the absorbed ezetimibe is converted to ezetimibe glucuronide (Patrick et al. 2002). The free ezetimibe to total ezetimibe *C*_max_ ratio in this study is 13.5% and 11.6% for reference and test treatment. These results support the rate of glucuronidation of ezetimibe. Glucuronide ezetimibe is responsible for pharmacological activity (Van Heek et al. 1997). This active metabolite contributes significantly to the cholesterol-lowering effect by inhibiting the absorption of dietary and biliary cholesterol at the brush border of the small intestine.

The study utilized a 4-period replicated design to address the high variability of telmisartan. This allowed for the assessment of ISV not only for the reference treatment but also for the test treatment. The ISV for telmisartan in the test treatment was found to be 44.4%. This is also similar to the ISV of 40.7% in the reference treatment.

Generally, HVDs with intrasubject variability exceeding 30% recommend a 3-period partially replicated design or a 4-period fully replicated design, applying the SABE approach (European Medicines Agency 2010; Ministry of Food and Drug Safety 2023). The ISV of telmisartan *C*_max_ in this study was 40.7%, and the 90% CI acceptance limits for the GMR using the SABE approach were 0.7284–1.3729.

Based on a previous study, telmisartan’s ISV was 42.94%, which indicated that 18 subjects would be sufficient to meet equivalent criteria with a 4-period replicated crossover design and the use of the SABE statistical approach (Kang et al. 2018). Also, previous studies have reported that ezetimibe exhibits an ISV of 34%, leading to a decision to recruit 58 participants with a 2-period crossover design. Despite this high variability, MFDS guidance (Ministry of Food and Drug Safety 2025) does not mandate a replicated study for equivalence assessment of ezetimibe. Therefore, a conventional 2 × 2 crossover design was adopted, and enough participants were recruited to evaluate the equivalence of telmisartan as well. Among the 49 subjects who completed all 4 periods of the study, the 90% CIs for telmisartan’s GMRs met equivalence criteria even without applying the SABE approach.

The established equivalence in pharmacokinetic characteristics of the FDC is significant in the context of combination therapy for chronic conditions. The results support its potential as a simplified treatment option for patients with both dyslipidemia and hypertension. Combining all three treatments into a single pill is expected to maximize the benefits of improving adherence, ultimately leading to better compliance and overall health outcomes.

Overall, the fixed-dose combination of ezetimibe, rosuvastatin, and telmisartan demonstrated comparable pharmacokinetic characteristics and safety profiles to the coadministration of separate tablets of these drugs.

## Data Availability

All source data for this work (or generated in this study) are available upon reasonable request.

## Abbreviations

ADR: Adverse drug reaction
AE: Adverse event
AUC_inf_: Area under the curve from dosing to infinity
AUC_t_: Area under the time-concentration curve to the last measurable concentration
BMI: Body mass index
CIs: Confidence intervals
CL/F: Apparent to total clearance
*C*_max_: Maximum plasma concentration
EMA: European Medicines Agency
FDC: Fixed-dose combination
GMR: Geometric mean ratio
HVD: Highly variable drug
IRB: Institutional Review Board
IS: Internal standard
ISV: Intra-subject variability
LDL-C: Low-density lipoprotein cholesterol
MFDS: Ministry of food and drug safety
MRM: Multiple reaction monitoring
PK: Pharmacokinetics
SABE: Scaled average bioequivalence
SAE: Serious adverse event
*t*_1/2_: Terminal elimination half-life
*T*_max_: Time to reach maximum plasma concentration
UPLC: Ultra-performance liquid chromatography
Vd/F: Apparent volume of distribution
